# ERα Agonist Protects Aged Female Mice From Sevoflurane Neurotoxicity via PTEN Nuclear Translocation

**DOI:** 10.1002/cns.70959

**Published:** 2026-05-29

**Authors:** Xudong Zhang, Yu Lei, Bingqing Gong, Yiquan Xu, Ding Li, Yonghao Yu, Feixiang Li, Jianghui Luo

**Affiliations:** ^1^ Department of Anesthesiology, Sichuan Cancer Hospital and Institute, Sichuan Cancer Center, School of Medicine University of Electronic Science and Technology of China Chengdu Sichuan China; ^2^ Department of Endoscopy Sichuan Cancer Hospital and Institute, Sichuan Cancer Center, School of Medicine University of Electronic Science and Technology of China Chengdu Sichuan China; ^3^ Department of Anesthesiology Tianjin Medical University General Hospital, Tianjin Institute of Anesthesiology Tianjin China; ^4^ Department of Anesthesiology, Beijing Chao‐Yang Hospital Capital Medical University Beijing China

**Keywords:** cognitive dysfunction, Erα, hippocampal synaptic plasticity, PTEN nuclear translocation, Sevoflurane

## Abstract

**Background:**

Repeated sevoflurane exposure has been associated with postoperative cognitive dysfunction, particularly in the aged population, yet the underlying mechanisms remain unclear. This study aimed to investigate whether PTEN nuclear translocation mediates sevoflurane‐induced hippocampal synaptic and cognitive deficits in aged female mice, and whether activation of ERα can mitigate these effects.

**Methods:**

Aged female mice were exposed to repeated sevoflurane, followed by assessment of PTEN subcellular localization using Western blot and immunofluorescence. Synaptic integrity was evaluated by PSD95 and SYP expression and dendritic spine density in the hippocampal CA1 region. Cognitive performance was assessed with the Morris water maze and Y‐maze tests. PTEN nuclear translocation was inhibited using AAV‐PTEN‐K13R, while Erα activation was achieved using PPT, and its effects were further evaluated in the presence of AAV‐shAagab, a regulator of PTEN nuclear translocation.

**Results:**

Repeated sevoflurane exposure significantly increased nuclear PTEN and decreased cytoplasmic PTEN, reduced PSD95 and synaptophysin expression, decreased dendritic spine density, and impaired spatial learning and working memory. Inhibition of PTEN nuclear translocation via AAV_PTEN‐K13R_ restored synaptic protein levels, spine density, and cognitive performance. Erα activation with PPT suppressed PTEN nuclear translocation, preserved synaptic structure, and improved cognitive function, whereas AAV_shAagab_ largely abolished these protective effects.

**Conclusion:**

Our study demonstrates that PTEN nuclear translocation mediates sevoflurane‐induced synaptic and cognitive deficits in aged female mice, and that Erα activation mitigates these effects by modulating PTEN localization. These findings suggest that Erα‐mediated regulation of PTEN may represent a potential therapeutic pathway for mitigating anesthesia‐related cognitive dysfunction in the elderly.

## Graphical abstract

Repeated sevoflurane exposure induces PTEN nuclear mislocalization, synaptic plasticity impairments, and cognitive deficits in aged female mice, whereas activation of the estrogen receptor α suppresses PTEN nuclear mislocalization, thereby ameliorating synaptic plasticity and cognitive dysfunction.

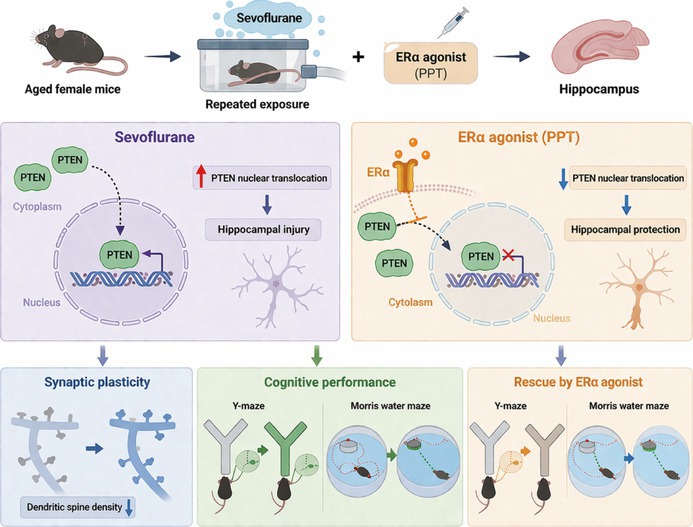

## 1 Introduction

Sevoflurane is one of the most commonly used volatile anesthetics in clinical practice due to its rapid induction, controllable depth, and favorable safety profile [1]. Despite these advantages, accumulating evidence indicates that sevoflurane can exert neurotoxic effects, particularly in the aging brain, where synaptic plasticity is naturally diminished [2, 3, 4]. In aged animals, sevoflurane exposure leads to dendritic spine loss, morphological alterations of spines, and synaptic ultrastructural disruption, resulting in persistent impairments in learning and memory [5, 6]. Mechanistically, these effects are closely associated with disturbances in calcium homeostasis, mitochondrial function, and activity‐dependent signaling, which collectively compromise long‐term potentiation and promote maladaptive synaptic remodeling [7]. Such alterations suggest that impaired synaptic plasticity is a central mechanism underlying sevoflurane‐induced cognitive deficits.

Phosphatase and tensin homolog (PTEN) is a well‐characterized tumor suppressor that also plays a critical role in the central nervous system [8]. In neurons, PTEN regulates dendritic growth, spine formation, and synaptic strength, contributing to the maintenance of circuit stability and neuronal homeostasis [9]. Beyond its cytoplasmic functions, PTEN can translocate to the nucleus in response to cellular stress or pathological stimuli. Nuclear PTEN has been implicated in the regulation of gene transcription, neuronal survival, and synaptic function, and aberrant nuclear accumulation has been observed in various neurological disorders characterized by synaptic dysfunction and cognitive impairment [10, 11]. Importantly, recent evidence has suggested that PTEN nuclear shuttling can be modulated by regulatory proteins such as Aagab, indicating that PTEN subcellular localization is dynamically controlled under pathological conditions [11]. Therefore, it is reasonable to speculate that in sevoflurane‐induced neurotoxicity, aberrant regulation of PTEN, particularly its nuclear accumulation, may be involved in mediating synaptic dysfunction.

Selective estrogen receptor alpha (Erα) agonists have emerged as potent neuroprotective agents, particularly in aged female subjects with reduced endogenous estrogen levels [12]. Activation of Erα enhances dendritic spine density, stabilizes synaptic structure, and improves cognitive outcomes in models of neurodegeneration and neurological injury [13, 14]. In addition to classical genomic effects, Erα agonists influence intracellular signaling, cytoskeletal dynamics, and protein trafficking, all of which are essential for synaptic maintenance [15, 16]. Notably, Erα activation may intersect with pathways regulating PTEN localization and function, raising the possibility that Erα agonists exert their neuroprotective effects by modulating PTEN nuclear translocation.

Based on these considerations, we hypothesized that a selective ERα agonist could mitigate sevoflurane‐induced neurotoxicity in aged female mice by preserving synaptic plasticity. This effect may be partly mediated through modulation of PTEN nuclear translocation, thereby attenuating anesthetic‐induced synaptic deficits.

## 2 Material and Methods

### 2.1 Animals

Female C57BL/6J aged mice (15–16 months of age) [17] were purchased from Vital River Laboratories (Beijing, China) and housed under standard laboratory conditions (22°C ± 2°C, 50% ± 10% humidity, 12‐h light/dark cycle) with ad libitum access to food and water. The animals were continuously housed under these conditions until they reached 15–16 months of age, at which point they were enrolled in the experimental procedures. All protocols were carried out in strict accordance with institutional guidelines for animal care and use and were approved by the appropriate ethics committee.

### 2.2 Sevoflurane Exposure and ERα Agonist Treatment

Following the protocol established in our previous studies [5, 18], aged female mice were exposed to sevoflurane to establish a neurotoxicity model. Over a period of three days, mice were maintained in a sealed chamber and exposed daily to 3% sevoflurane in 60% oxygen for 2 h. The chamber temperature was maintained at 37°C, and the concentrations of oxygen and sevoflurane were continuously monitored throughout the exposure period. After each session, mice were returned to their home cages for recovery under standard laboratory conditions.

Thirty minutes prior to each sevoflurane exposure, mice were randomly assigned to receive either the selective ERα agonist propylpyrazole‐triol (PPT, 1 mg/kg) or an equivalent volume of vehicle via intraperitoneal injection. This treatment regimen was applied consistently across all exposure days [19]. Following completion of the treatment protocol, animals underwent behavioral testing, biochemical analyses, and histological assessments to evaluate the neuroprotective effects of PPT.

### 2.3 Western Blotting

Protein expression was assessed by immunoblotting using pre‐cast polyacrylamide gels (GenScript, Nanjing, China). Mice were deeply anesthetized, and hippocampal tissues were rapidly dissected on ice. For total protein extraction, tissues were homogenized in ice‐cold lysis buffer supplemented with protease and phosphatase inhibitors, followed by centrifugation to remove insoluble material. The supernatants were collected, and protein concentrations were determined using a bicinchoninic acid assay.

For nuclear and cytoplasmic fractionation, hippocampal tissues were homogenized in ice‐cold cytoplasmic extraction buffer containing protease and phosphatase inhibitors. After incubation on ice, samples were centrifuged at low speed to separate the cytoplasmic supernatant from the nuclear pellet. The supernatant was further clarified by high‐speed centrifugation to obtain the cytoplasmic fraction. The nuclear pellet was washed and subsequently lysed in nuclear extraction buffer with intermittent mixing, followed by centrifugation to collect the nuclear protein fraction.

Equal amounts of protein were separated by SDS–PAGE and transferred onto PVDF membranes. Membranes were blocked and incubated overnight at 4°C with primary antibodies (PTEN, 1:1000, Abcam; Lamin B, 1:1000, Proteintech; PSD95, 1:5000, Proteintech; SYP, 1:5000, Proteintech; GAPDH/β‐actin, 1:5000, Affinity). After washing, membranes were incubated with appropriate horseradish peroxidase–conjugated secondary antibodies. Protein bands were visualized using an enhanced chemiluminescence system. Band intensities were quantified using ImageJ software and normalized to the corresponding loading controls.

### 2.4 Immunofluorescence

Mice were deeply anesthetized, and hippocampal tissues were promptly isolated and processed for immunofluorescence analysis. Briefly, brains were fixed in paraformaldehyde, cryoprotected in graded sucrose solutions, and sectioned coronally using a cryostat. Free‐floating sections containing the hippocampus were collected for subsequent staining procedures.

Sections were permeabilized and incubated in blocking solution to reduce nonspecific binding, followed by incubation with primary antibodies (PTEN, 1:200, ThermoFisher Scientific; H3, 1:200, Millipore) at 4°C overnight. After thorough rinsing, sections were exposed to species‐appropriate fluorescent secondary antibodies under light‐protected conditions. Nuclei were counterstained with DAPI. Stained sections were mounted with antifade medium and imaged using a fluorescence microscope or confocal imaging system. All images were acquired using identical acquisition settings across experimental groups.

### 2.5 Golgi Staining

Neuronal morphology was evaluated using a Golgi staining kit (BD Biosciences, USA) with minor optimization. Mice were deeply anesthetized, and fresh brains were rapidly removed without fixation. Hippocampal tissue blocks (< 5 mm thick) were immediately immersed in freshly prepared impregnation solution consisting of equal volumes of Solutions A and B and incubated in the dark at room temperature for 21 days. The solution was replaced with a fresh impregnation solution after 14 days to ensure uniform and optimal staining.

After impregnation, tissues were transferred into Solution C for protection and stored at 4°C in the dark for a minimum of 72 h. Coronal sections were cut with a vibratome, mounted on gelatin‐coated slides, and air‐dried. A staining working solution was freshly prepared by mixing Solutions D and E with distilled water according to the manufacturer's instructions and applied to fully cover the sections for 10 min at room temperature in the dark. Sections were then rinsed 3–4 times with distilled water, dehydrated through graded ethanol, cleared in xylene, and coverslipped with neutral mounting medium. After drying, hippocampal neurons with intact somata, clearly defined dendritic arbors, and visible spines were selected for quantitative morphological analysis, and only third‐order (tertiary) dendritic branches were used for spine evaluation.

### 2.6 Morris Water Maze

Spatial learning and memory were evaluated using the Morris Water Maze (MWM) over 5 consecutive days. The circular pool was filled with opaque water at 22°C–25°C, and a hidden platform was submerged 1–2 cm below the water surface. Distal visual cues were placed around the pool to facilitate navigation.

The experiment consisted of a training phase and a test phase. During the training phase, mice were released from different quadrants in a pseudo‐random order. Each trial lasted until the mouse reached the platform or 60 s elapsed, after which the animal remained on the platform for 15 s. Escape latency, swim path length, and swimming speed were recorded using an automated tracking system over the 5‐day training period. The test phase was conducted 1 h after the final training session on day 5, with the platform removed. Memory retention was evaluated by counting the number of crossings over the former platform location and measuring the time spent in the target quadrant.

### 2.7 Y Maze

The Y‐maze spontaneous alternation task, consisting of three identical arms (30 × 8 × 15 cm) arranged at 120° angles, was used to assess working memory. The floor and walls were made of non‐reflective material, and distal visual cues were positioned around the maze to aid spatial orientation.

The experiment was divided into a training phase and a test phase. During the training phase, one arm was designated as the familiar arm and another as the initial arm, while the third arm remained closed. Mice were placed in the initial arm and allowed to freely explore the open arms for 10 min. During the test phase, all three arms were opened, and mice were allowed to explore freely. Behavioral metrics, including total distance traveled, number of entries into the novel arm, and time spent in the novel arm, were recorded using an automated tracking system. The maze was cleaned with 70% ethanol between trials to eliminate olfactory cues.

All behavioral tests were conducted 3 weeks after completion of the sevoflurane exposure.

### 2.8 Stereotaxic Viral Injection

Stereotaxic injections were performed as previously described. Briefly, mice were anesthetized with isoflurane and placed in a stereotaxic apparatus. Recombinant adeno‐associated viruses carrying AAV_PTEN‐K13R_, AAV_shAagab_, or the corresponding control vehicle (AAV‐Vehicle) (customized by OBiO Technology, Shanghai, China) were used for viral delivery. The plasmid sequences used for viral construction were obtained from previously published studies [11]. Validation of the viral constructs is shown in Figure S1. The viruses were bilaterally injected into the hippocampus using the following stereotaxic coordinates relative to bregma (AP −1.85 mm, ML ±1.5 mm, DV −1.8 mm).

A total volume of 0.5 μL viral suspension was delivered per site using a Hamilton microsyringe at a rate of 0.3 μL/min. After injection, the needle was kept in place for an additional 5 min to allow diffusion of the viral particles and to minimize reflux along the injection tract, and then slowly withdrawn. All viral injections were performed 2 weeks prior to sevoflurane exposure. Following surgery, mice were maintained on a heating pad until full recovery from anesthesia and subsequently returned to their home cages.

### 2.9 Statistical Analysis

Data are reported as mean values ± standard error of the mean (SEM). Normality of each dataset was first evaluated using the Shapiro–Wilk test. For datasets following a normal distribution, comparisons between two groups were conducted using unpaired Student's *t*‐tests, whereas experiments involving multiple factors were analyzed by one‐way or two‐way ANOVA, followed by post hoc tests (Tukey's or Bonferroni) to account for multiple comparisons.

For datasets that deviated from normality, nonparametric approaches were applied. Specifically, two‐group comparisons employed the Mann–Whitney U test, and multiple‐group comparisons used the Kruskal–Wallis test with Dunn's post hoc correction. Differences were considered statistically significant at *p* < 0.05. Significance levels are defined as: ns (not statistically significant), *p < 0.05, **p < 0.01, ***p < 0.001. All “n” values represent independent biological replicates per group.

## 3 Results

### 3.1 Repeated Sevoflurane Exposure Promotes PTEN Nuclear Translocation and Cognitive Impairment

**FIGURE 1:**
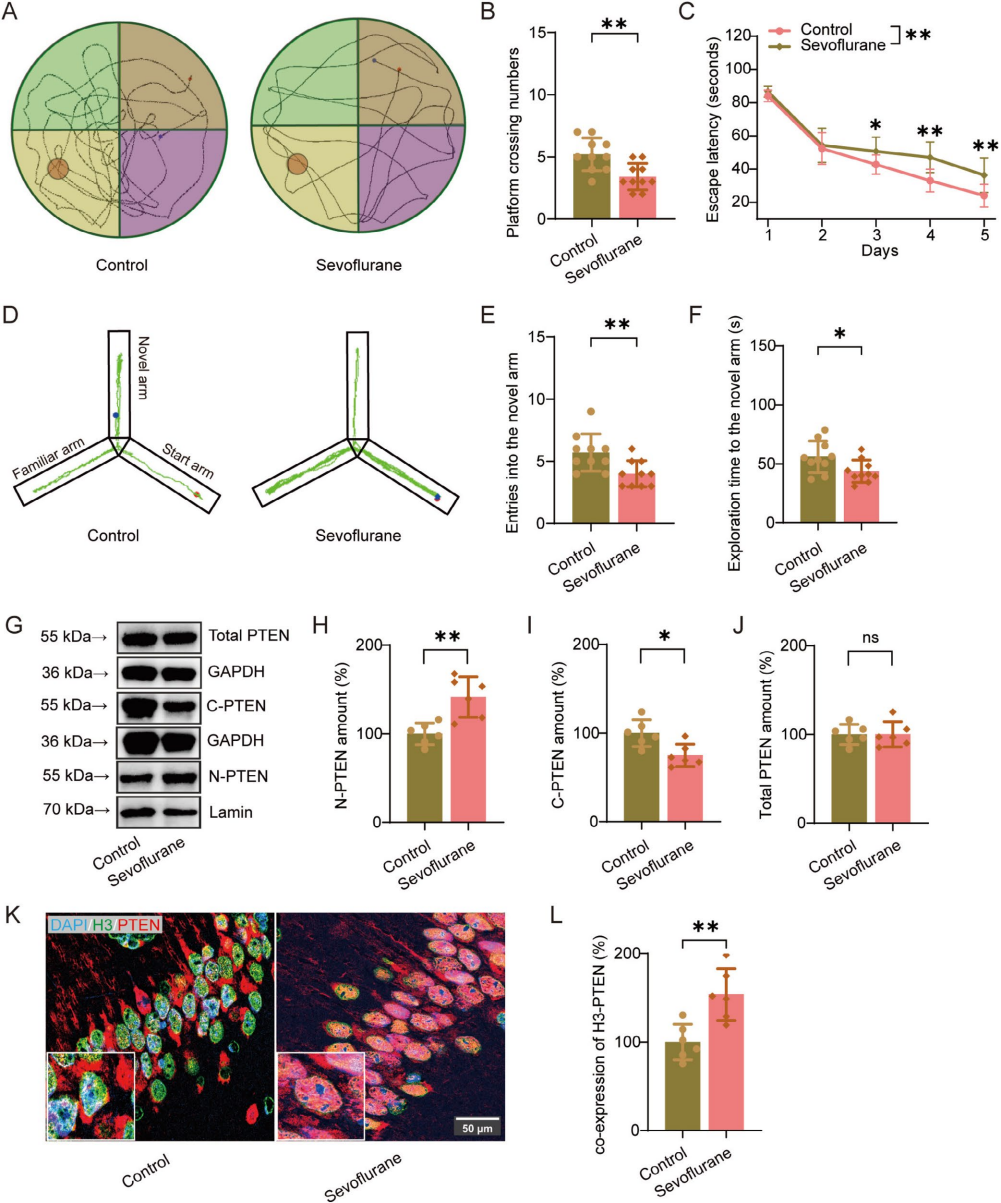
Repeated sevoflurane exposure increases PTEN nuclear localization and impairs cognitive function. (A) Representative navigation trajectories of mice in the probe trial of the Morris water maze. (B, C) Number of passes over the previous platform location (B) and time mice spent in the target quadrant (C) (*n* = 10/group). (D) Representative movement trajectories of mice in the Y‐maze test. (E, F) Frequency of entries into the novel arm (E) and duration spent in the novel arm (F) (*n* = 10/group). (G) Representative Western blot images showing total PTEN, nuclear PTEN, and cytoplasmic PTEN levels in the hippocampus. (H–J) Quantification of nuclear PTEN (H), cytoplasmic PTEN (I), and total PTEN (J) protein levels (*n* = 6/group). (K) Hippocampal immunofluorescence images depicting PTEN (red) and the nuclear marker H3 (green) in the hippocampus. Nuclei were counterstained with DAPI (blue). (L) Quantification of PTEN fluorescence intensity within the nucleus (*n* = 6/group). cytoplasmic PTEN (C‐PTEN); nuclear PTEN (N‐PTEN).

We first assessed the impact of repeated sevoflurane exposure on cognitive function. Behavioral assessments demonstrated that repeated sevoflurane exposure markedly impaired both spatial and working memory. In the MWM, exposed mice displayed fewer crossings over the hidden platform (Figure 1A,B; t(18) = 3.349, *p =* 0.004) and a markedly increased escape latency (Figure 1A,C; F(1,18) = 14.357, *p* = 0.001). Similarly, Y‐maze testing revealed a reduction in entries into the novel arm (Figure 1D,E; t(18) = 2.94, *p =* 0.009) and shorter exploration duration (Figure 1D,F; t(18) = 2.378, *p =* 0.029), indicating compromised working memory and exploratory behavior.

To investigate whether sevoflurane exposure influences PTEN nuclear translocation, we analyzed the subcellular distribution of PTEN in nuclear and cytoplasmic fractions by Western blotting. The results showed that repeated sevoflurane exposure markedly increased PTEN levels in the nuclear fraction of the hippocampus (Figure 1G,H; t(10) = −3.927, *p =* 0.003), accompanied by a corresponding decrease in cytoplasmic PTEN. Notably (Figure 1G,I; t(10) = 3.103, *p =* 0.011), the total PTEN protein level remained unchanged (Figure 1G,J; t(10) = −0.023, *p =* 0.982). To further confirm these findings, immunofluorescence staining was used to assess the subcellular distribution of PTEN in the hippocampal CA1 region. Consistent with the Western blot results, repeated sevoflurane exposure markedly increased PTEN levels in the nucleus (Figure 1K,L; t(10) = −3.71, *p =* 0.004). Collectively, these findings suggest that PTEN nuclear translocation may represent a key molecular mechanism contributing to sevoflurane‐induced hippocampal dysfunction and associated cognitive impairment.

### 3.2 PTEN Nuclear Translocation Mediates Sevoflurane‐Induced Synaptic Plasticity Alterations

**FIGURE 2:**
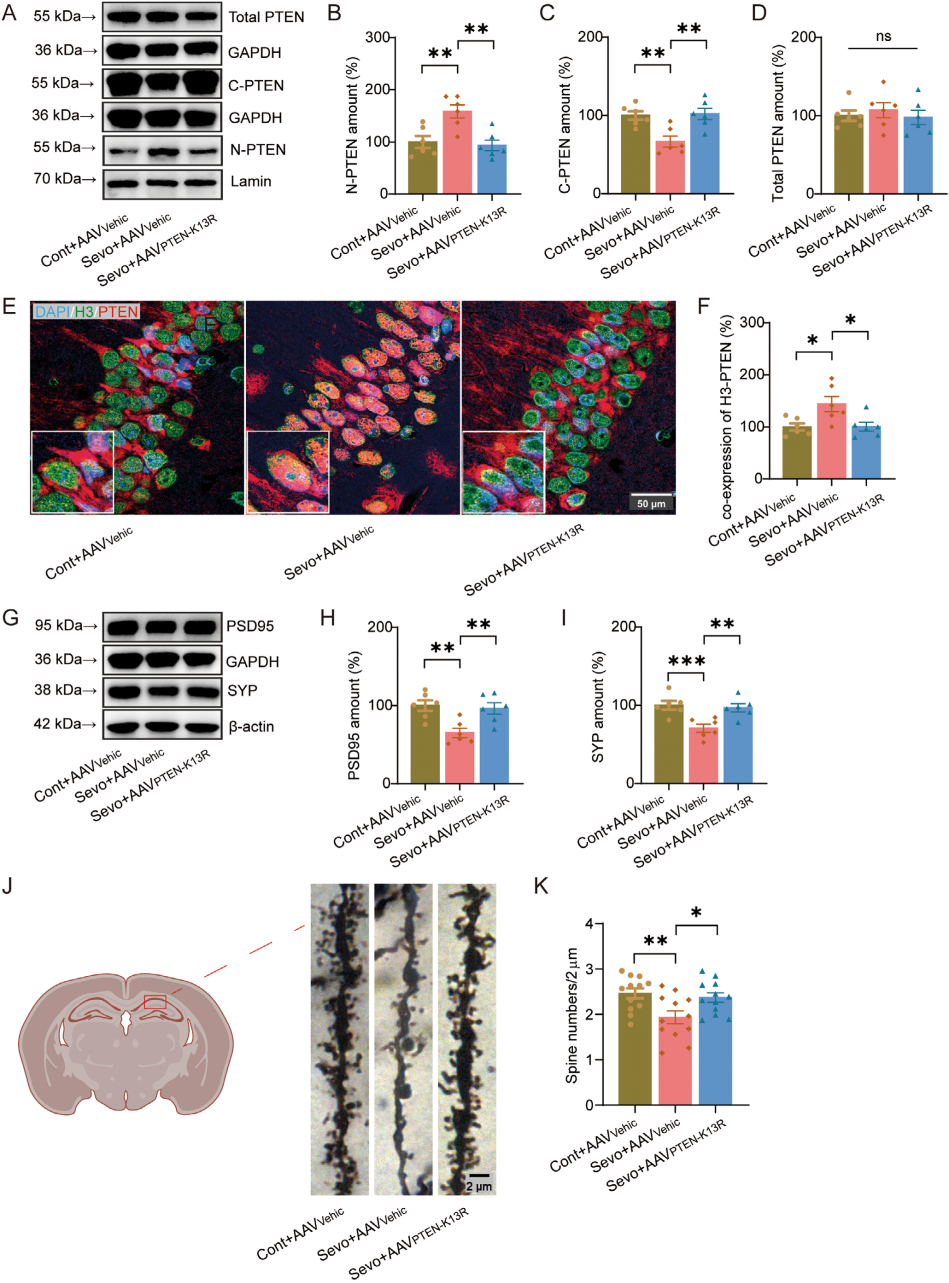
PTEN nuclear translocation mediates sevoflurane‐induced synaptic plasticity alterations. (A) Representative Western blot images showing total PTEN, nuclear PTEN, and cytoplasmic PTEN levels in the hippocampus. (B–D) Quantification of nuclear PTEN (B), cytoplasmic PTEN (C), and total PTEN (D) protein levels (*n* = 6/group). (E) Hippocampal immunofluorescence images depicting PTEN (red) and the nuclear marker H3 (green) in the hippocampus. Nuclei were counterstained with DAPI (blue). (F) Quantification of PTEN fluorescence intensity within the nucleus (*n* = 6/group). (G) Representative Western blot images showing the synaptic proteins PSD95 and SYP in the hippocampus. (H, I) Quantification of PSD95 (H) and SYP (I) protein levels (*n* = 6/group). (J) Representative images of dendritic spines in the hippocampal CA1 region. (K) Quantification of dendritic spine density (*n* = 6/group). cytoplasmic PTEN (C‐PTEN); nuclear PTEN (N‐PTEN); Control (Cont); Vehicle (Vehic); Sevoflurane (Sevo).

To further determine the role of PTEN nuclear translocation in sevoflurane‐induced neurotoxicity, we utilized an adeno‐associated virus carrying a PTEN point mutation that inhibits its nuclear translocation (AAV_PTEN‐K13R_). Western blot analysis showed that sevoflurane markedly increased PTEN levels in the nuclear fraction of the hippocampus (Figure 2A,B; t(15) = −3.652, *p =* 0.002) while concomitantly reducing cytoplasmic PTEN (Figure 2A,C; t(15) = 3.594, *p =* 0.003), without altering total PTEN expression (Figure 2A,D; F(2,15) = 0.324, *p =* 0.728). Importantly, AAV_PTEN‐K13R_ effectively prevented this sevoflurane‐induced nuclear accumulation of PTEN (t(15) = 4.057, *p =* 0.001) and restored its cytoplasmic distribution (t(15) = −3.804, *p =* 0.002).

Consistent with the WB results, immunofluorescence analysis further revealed a pronounced redistribution of PTEN from the cytoplasm to the nucleus in the hippocampal CA1 region following sevoflurane anesthesia (Figure 2E,F; t(15) = −2.966, *p =* 0.01). In contrast, inhibition of PTEN nuclear translocation by AAV_PTEN‐K13R_ largely normalized this abnormal subcellular localization (t(15) = 2.936, *p =* 0.01).

We next investigated whether PTEN nuclear translocation contributes to sevoflurane‐induced synaptic alterations. Western blot analysis demonstrated that sevoflurane significantly reduced the expression of the synaptic proteins PSD95 (Figure 2G,H; t(15) = 3.687, *p =* 0.002) and SYP (Figure 2G,I; t(15) = 3.947, *p =* 0.001), suggesting impaired synaptic integrity. Notably, inhibition of PTEN nuclear translocation by AAV_PTEN‐K13R_ largely abolished these reductions (PSD95, t(15) = −3.288, *p =* 0.005; SYP, t(15) = −3.503, *p =* 0.003).

To further assess structural synaptic changes, Golgi staining was performed to examine dendritic spine morphology in the hippocampal CA1 region. Sevoflurane treatment resulted in a significant decrease in dendritic spine density (Figure 2J,K; t(33) = 3.126, *p =* 0.004), whereas AAV_PTEN‐K13R_ administration effectively rescued this deficit (t(33) = −2.564, *p =* 0.015).

Collectively, these results indicate that PTEN nuclear translocation is a critical mediator of sevoflurane‐induced synaptic plasticity impairment in the hippocampus.

### 3.3 PTEN Nuclear Translocation Mediates Sevoflurane‐Induced Cognitive Impairment

**FIGURE 3:**
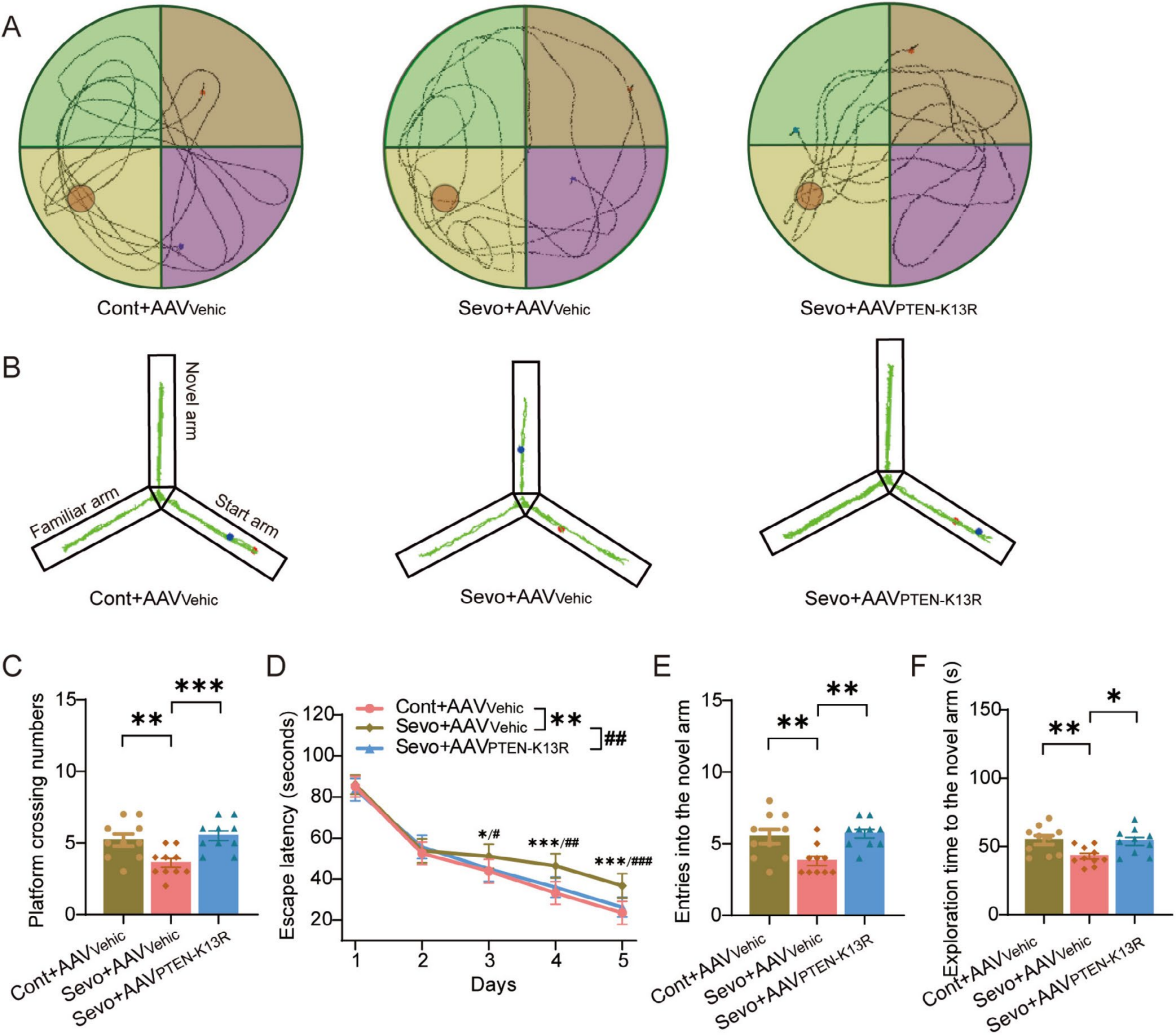
PTEN nuclear translocation mediates sevoflurane‐induced cognitive impairment. (A) Representative navigation trajectories of mice in the probe trial of the Morris water maze. (B) Representative movement trajectories of mice in the Y‐maze test. (C, D) Number of passes over the previous platform location (C) and time mice spent in the target quadrant (D) (*n* = 10/group). (E, F) Frequency of entries into the novel arm (E) and duration spent in the novel arm (F) (*n* = 10/group). Control (Cont); Vehicle (Vehic); Sevoflurane (Sevo).

To further determine whether PTEN nuclear translocation contributes to sevoflurane‐induced cognitive deficits, we next evaluated cognitive performance using behavioral assays. In the MWM, sevoflurane anesthesia significantly reduced the number of crossings over the hidden platform (Figure 3A,C; t(27) = 3.165, *p =* 0.004) and markedly increased the escape latency (Figure 3A,D; F(1, 18) = 11.083, *p* = 0.004). Notably, inhibition of PTEN nuclear translocation by AAV_PTEN‐K13R_ markedly reversed these deficits (Crossings: t(27) = −3.759, *p =* 0.001; Latency: F(1, 18) = 9.607, *p* = 0.006).

Consistently, Y‐maze testing showed that sevoflurane exposure significantly decreased both the number of entries into the novel arm (Figure 3B,E; t(27) = 3.115, *p =* 0.004) and the time spent exploring it (Figure 3B,F; t(27) = 2.98, *p =* 0.006), indicating impaired working memory. Importantly, AAV_PTEN‐K13R_ treatment effectively restored these behavioral impairments induced by sevoflurane (Number of entries: t(27) = −3.482, *p =* 0.002; Exploration time: t(27) = −2.733, *p =* 0.011).

Collectively, these behavioral findings indicate that PTEN nuclear translocation mediates the cognitive dysfunction induced by sevoflurane anesthesia.

### 3.4 Estrogen Receptor α Agonist Inhibits PTEN Nuclear Translocation and Restores Hippocampal Synaptic Plasticity

Previous studies have demonstrated that activation of ERα exerts neuroprotective effects. We therefore investigated whether pharmacological activation of ERα using PPT could mitigate sevoflurane‐induced neurotoxicity and regulate PTEN nuclear translocation. To further clarify the role of PTEN nuclear translocation in this process, an adeno‐associated virus targeting Aagab (AAV_shAagab_), which has been reported to facilitate PTEN nuclear translocation, was employed.

**FIGURE 4:**
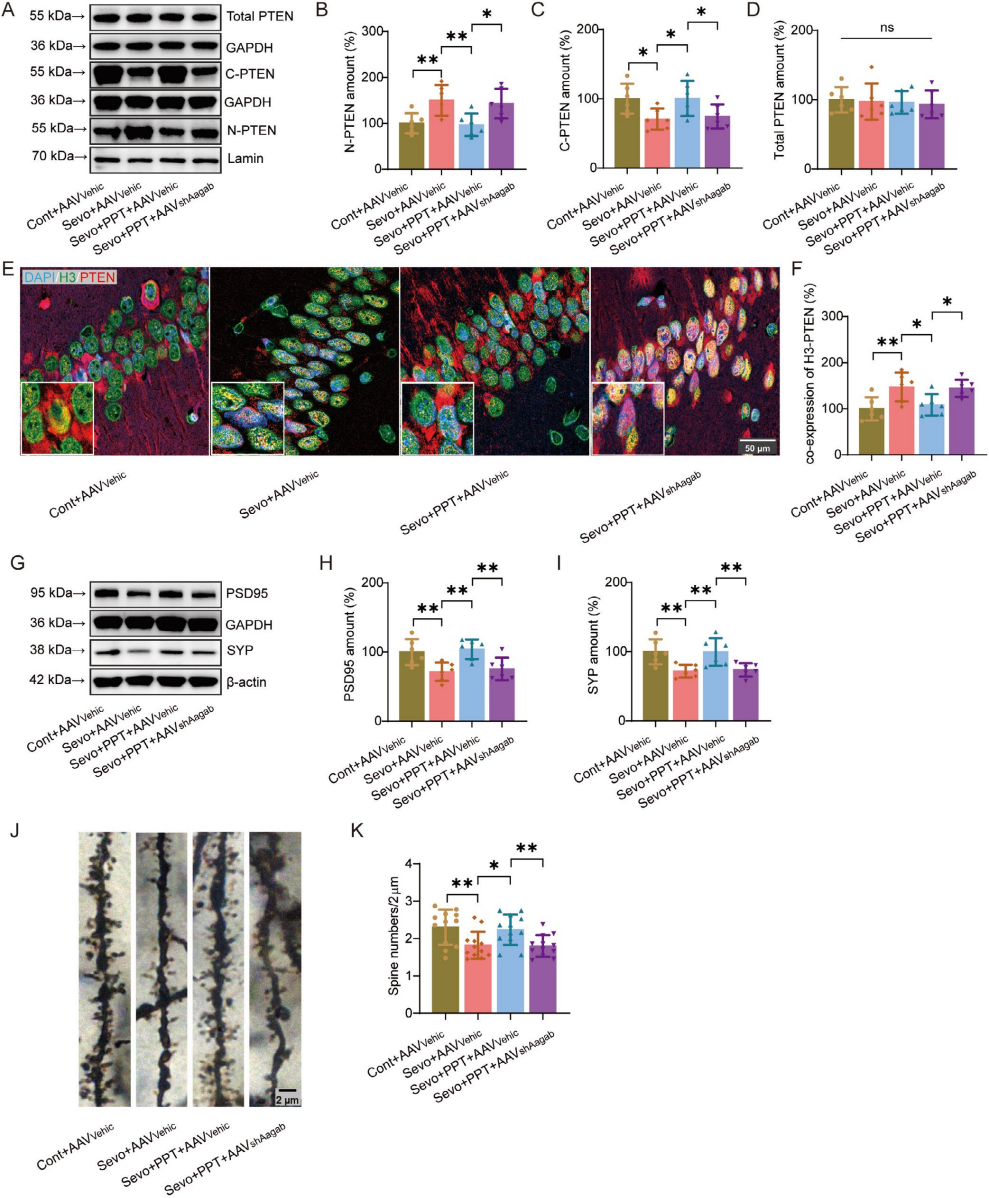
Estrogen receptor α agonist inhibits PTEN nuclear translocation and restores hippocampal synaptic plasticity. (A) Representative Western blot images showing total PTEN, nuclear PTEN, and cytoplasmic PTEN levels in the hippocampus. (B–D) Quantification of nuclear PTEN (B), cytoplasmic PTEN (C), and total PTEN (D) protein levels (*n* = 6/group). (E) Hippocampal immunofluorescence images depicting PTEN (red) and the nuclear marker H3 (green) in the hippocampus. Nuclei were counterstained with DAPI (blue). (F) Quantification of PTEN fluorescence intensity within the nucleus (*n* = 6/group). (G) Representative Western blot images showing the synaptic proteins PSD95 and SYP in the hippocampus. (H, I) Quantification of PSD95 (H) and SYP (I) protein levels (*n* = 6/group). (J) Representative images of dendritic spines in the hippocampal CA1 region. (K) Quantification of dendritic spine density (*n* = 6/group). cytoplasmic PTEN (C‐PTEN); nuclear PTEN (N‐PTEN); Control (Cont); Vehicle (Vehic); Sevoflurane (Sevo).

Western blot analysis showed that PPT treatment markedly reduced PTEN levels in the nuclear fraction of the hippocampus (Figure 4A,B; t(20) = 3.253, *p =* 0.004) while concomitantly increasing cytoplasmic PTEN (Figure 4A,C; t(20) = −2.531, *p =* 0.02). Notably, AAV_shAagab_ administration largely reversed the inhibitory effect of PPT on PTEN nuclear translocation (N‐PTEN: t(20) = −2.816, *p =* 0.011; C‐PTEN: t(20) = −2.205, *p =* 0.039).

Consistent with the WB results, immunofluorescence staining further demonstrated that PPT significantly suppressed the sevoflurane‐induced redistribution of PTEN from the cytoplasm to the nucleus in the hippocampal CA1 region (Figure 4E,F; t(20) = 2.674, *p =* 0.015). In contrast, AAV_shAagab_ markedly attenuated this effect and restored PTEN nuclear accumulation (t(20) = −2.488, *p =* 0.022).

To determine whether these molecular alterations were associated with synaptic changes, we next examined the expression of the synaptic marker proteins PSD95 and SYP. PPT treatment significantly increased the levels of PSD95 (Figure 4G,H; t(20) = −3.601, *p =* 0.002) and SYP (Figure 4G,I; t(20) = −3.169, *p =* 0.005), whereas AAV_shAagab_ administration largely abolished this protective effect (PSD95: t(20) = 3.124, *p =* 0.005; SYP: t(20) = 2.981, *p =* 0.007).

Furthermore, dendritic spine morphology in the hippocampal CA1 region was assessed using Golgi staining. PPT markedly increased dendritic spine density (Figure 4J,K; t(44) = −2.609, *p =* 0.012), whereas AAV_shAagab_ treatment reversed this effect (t(44) = −2.74, *p =* 0.009).

Taken together, these findings suggest that Erα activation alleviates sevoflurane‐induced synaptic impairment, at least in part, by inhibiting PTEN nuclear translocation.

### 3.5 ERα Agonist Alleviates Sevoflurane‐Induced Cognitive Impairment

**FIGURE 5:**
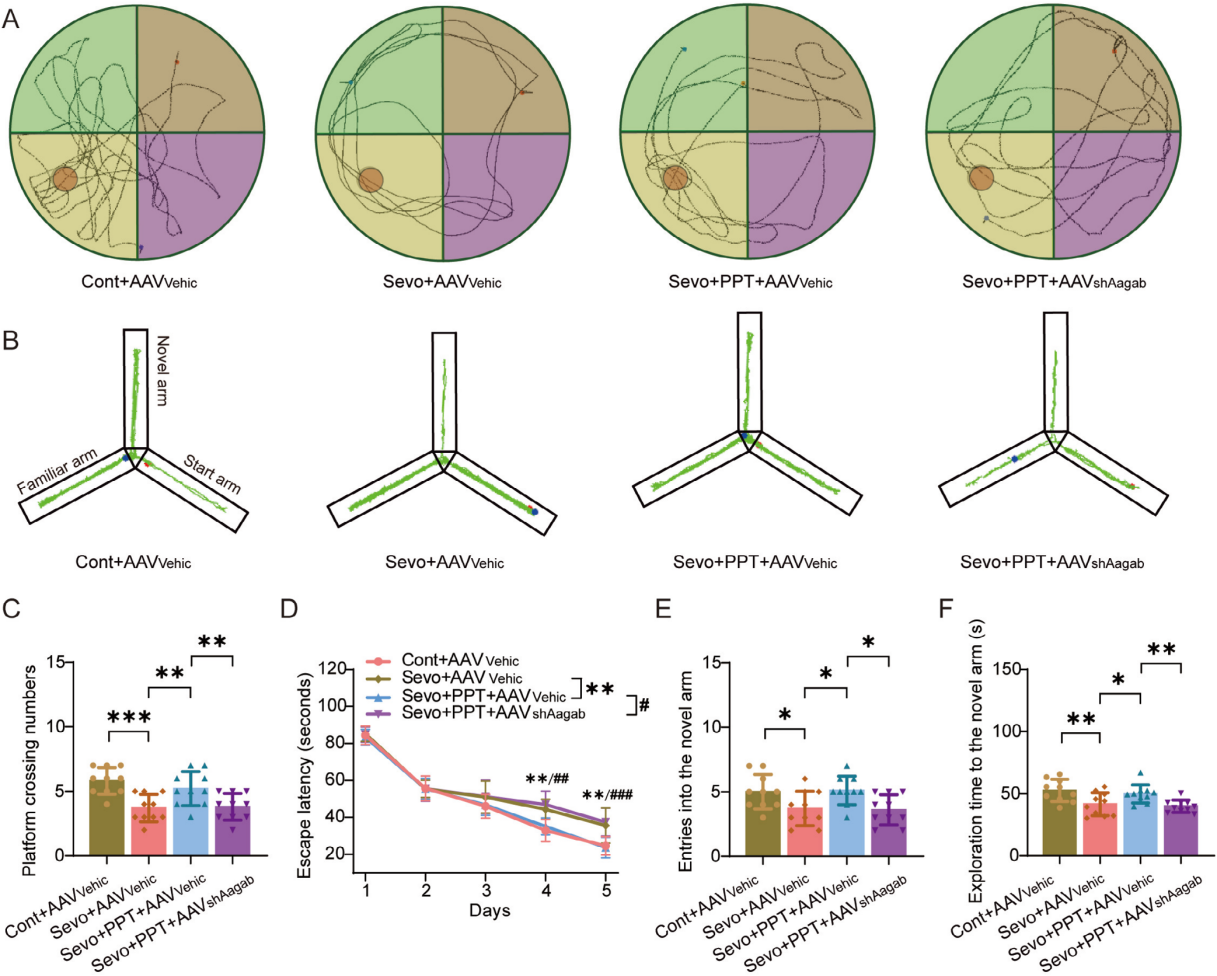
ERα agonist alleviates sevoflurane‐induced cognitive impairment. (A) Representative navigation trajectories of mice in the probe trial of the Morris water maze. (B) Representative movement trajectories of mice in the Y‐maze test. (C, D) Number of passes over the previous platform location (C) and time mice spent in the target quadrant (D) (*n* = 10/group). (E, F) Frequency of entries into the novel arm (E) and duration spent in the novel arm (F) (*n* = 10/group). Control (Cont); Vehicle (Vehic); Sevoflurane (Sevo).

We further investigated the effects of PPT on sevoflurane‐induced cognitive impairment. In the MWM, PPT treatment significantly increased the number of platform crossings (Figure 5A,C; t(36) = −3.003, *p =* 0.005) and markedly increased the escape latency (Figure 5A,D; F(1, 18) = 13.528, *p* = 0.002). Notably, these improvements were largely abolished by AAV_shAagab_ administration (Crossings: t(36) = 2.803, *p =* 0.008; Latency: F(1, 18) = 8.047, *p* = 0.011).

Consistently, the Y‐maze test showed that PPT treatment significantly increased both the number of entries into the novel arm (Figure 5B,E; t(36) = −2.523, *p =* 0.016) and the time spent exploring the novel arm (Figure 5B,F; t(36) = −2.352, *p =* 0.024), whereas AAV_shAagab_ reversed these effects (Number of entries: t(36) = 2.704, *p =* 0.01; Exploration time: t(36) = 2.827, *p =* 0.008).

Taken together, these behavioral results indicate that activation of ERα by PPT effectively alleviates sevoflurane‐induced cognitive impairment.

## 4 Discussion

In the present study, we demonstrated that repeated sevoflurane exposure induces significant cognitive impairment in aged mice, which is accompanied by abnormal nuclear translocation of PTEN and synaptic dysfunction in the hippocampus. Mechanistically, inhibition of PTEN nuclear translocation using AAV_PTEN‐K13R_ effectively alleviated sevoflurane‐induced synaptic deficits and cognitive impairment. Furthermore, pharmacological activation of ERα by PPT suppressed PTEN nuclear translocation and improved both synaptic plasticity and behavioral performance. Importantly, these protective effects were largely abolished by AAV_shAagab_, suggesting that PTEN nuclear translocation plays a critical role in mediating sevoflurane‐induced neurotoxicity and the neuroprotective actions of ERα activation.

Synaptic plasticity is widely recognized as the cellular basis of learning and memory [20, 21]. Structural and functional alterations in synapses are closely associated with cognitive deficits [22, 23]. In aged mice, repeated sevoflurane exposure significantly reduced the expression of the synaptic proteins PSD95 and SYP, accompanied by decreased dendritic spine density in the hippocampal CA1 region. Our previous study further showed that repeated sevoflurane exposure significantly increases the number of TUNEL‐positive neurons in the hippocampus [5]. These findings indicate that sevoflurane exposure leads to pronounced synaptic impairment, which likely contributes to the observed cognitive dysfunction in the aged brain.

PTEN is a critical regulator of neuronal signaling and synaptic plasticity [24, 25]. In addition to its well‐known cytoplasmic functions, emerging evidence suggests that PTEN can shuttle between the cytoplasm and the nucleus, and its nuclear localization may influence neuronal survival and synaptic regulation [26, 27]. Our results showed that repeated sevoflurane exposure selectively altered the subcellular distribution of PTEN in the hippocampus, enhancing nuclear accumulation and diminishing cytoplasmic levels, without altering total PTEN expression. These findings suggest that sevoflurane primarily affects the intracellular distribution of PTEN rather than its overall expression. In addition, PTEN nuclear signaling may indirectly modulate synaptic function through crosstalk with PI3K/AKT‐dependent pathways, which are critical for dendritic spine stability and synaptic protein synthesis [28]. Therefore, it is plausible that sevoflurane‐induced nuclear accumulation of PTEN may disrupt these downstream signaling networks, thereby potentially contributing to impaired synaptic integrity and cognitive decline. Importantly, inhibition of PTEN nuclear translocation using AAV_PTEN‐K13R_ effectively restored synaptic protein expression, dendritic spine density, and cognitive performance, demonstrating that PTEN nuclear translocation is a key mechanism underlying sevoflurane‐induced synaptic and cognitive deficits in the aged brain. However, whether synaptic dysfunction may in turn influence PTEN nuclear translocation remains to be further investigated in future studies.

Estrogen receptors, particularly ERα, play important roles in maintaining neuronal function and synaptic plasticity, and their neuroprotective effects may be particularly relevant in aging [29, 30]. Previous studies have shown that activation of ERα exerts neuroprotective effects in various neurological disorders [31, 32]. In the present study, pharmacological activation of ERα with PPT significantly suppressed sevoflurane‐induced PTEN nuclear translocation, increased synaptic protein expression, and restored dendritic spine density in the hippocampus of aged mice. Moreover, PPT treatment improved behavioral performance in both the Morris water maze and Y‐maze tests, indicating a substantial protective effect against sevoflurane‐induced cognitive impairment in the aged brain. Notably, the beneficial effects of PPT were largely abolished by AAV_shAagab_, which promotes PTEN nuclear translocation. This finding further supports the notion that inhibition of PTEN nuclear translocation is an important mechanism through which ERα activation exerts its neuroprotective effects in aged mice. Taken together, these results suggest that ERα signaling may regulate hippocampal synaptic plasticity and cognitive function in aging by modulating the subcellular localization of PTEN.

Several limitations of this study should also be acknowledged. First, although our results demonstrate a critical role of PTEN nuclear translocation in sevoflurane‐induced neurotoxicity, the downstream signaling pathways involved remain to be fully elucidated. Second, the present study focused primarily on hippocampal synaptic alterations in aged mice, while other brain regions involved in cognitive regulation were not examined. Third, the study was conducted exclusively in aged female mice; therefore, whether similar mechanisms are present in aged male mice remains unclear and warrants further investigation. In addition, potential species differences between rodent models and humans should be considered when extrapolating our findings to clinical postoperative cognitive dysfunction. Although we observed changes in PTEN nuclear translocation, we did not directly assess potential alterations in PTEN functional activity, which may represent an additional layer of regulation to be explored in future studies.

In summary, our study demonstrates that repeated sevoflurane exposure induces hippocampal synaptic deficits and cognitive impairment in aged mice via enhanced PTEN nuclear translocation. Pharmacological activation of ERα effectively mitigates PTEN nuclear accumulation and substantially alleviates sevoflurane‐induced synaptic and cognitive impairments. These findings suggest a potential mechanism underlying sevoflurane‐induced neurotoxicity in the aged brain and indicate that ERα‐mediated regulation of PTEN may be involved in the associated synaptic and cognitive impairments in elderly populations.

## Funding

This work was supported by the Beijing Chao‐Yang Hospital Golden Seeds Foundation, CYJZ202523; the National Natural Science Foundation of China, 82572460; and the Tianjin Key Medical Discipline (Specialty) Construction Project, TJYXZDXK‐036A.

## Ethics Statement

All animal experiments were approved by the Animal Ethics Committee of Tianjin Medical University (Approval No. B2024‐DW‐61) and conducted in accordance with relevant institutional guidelines.

## Consent

All authors contributed to and have approved the final manuscript.

## Conflicts of Interest

The authors declare no conflicts of interest.

## Supporting information


**Figure S1:** cns70959‐sup‐0001‐FigureS1.tif. **Validation of Viral Constructs**. (A) Schematic illustration and titer validation of the AAVPTEN‐K13R construct. (B) Representative Western blot images and corresponding quantitative analysis showing protein knockdown efficiency of AAVshAagab (*n* = 10/group).

## Data Availability

The data that support the findings of this study are available from the corresponding author upon reasonable request.
