# Supplementary figures and images for: ERα Agonist Protects Aged Female Mice From Sevoflurane Neurotoxicity via PTEN Nuclear Translocation
Source: CNS Neurosci Ther. 2026 May 29;32(6):e70959. doi: 10.1002/cns.70959 (PMC13239482; doi:10.1002/cns.70959)

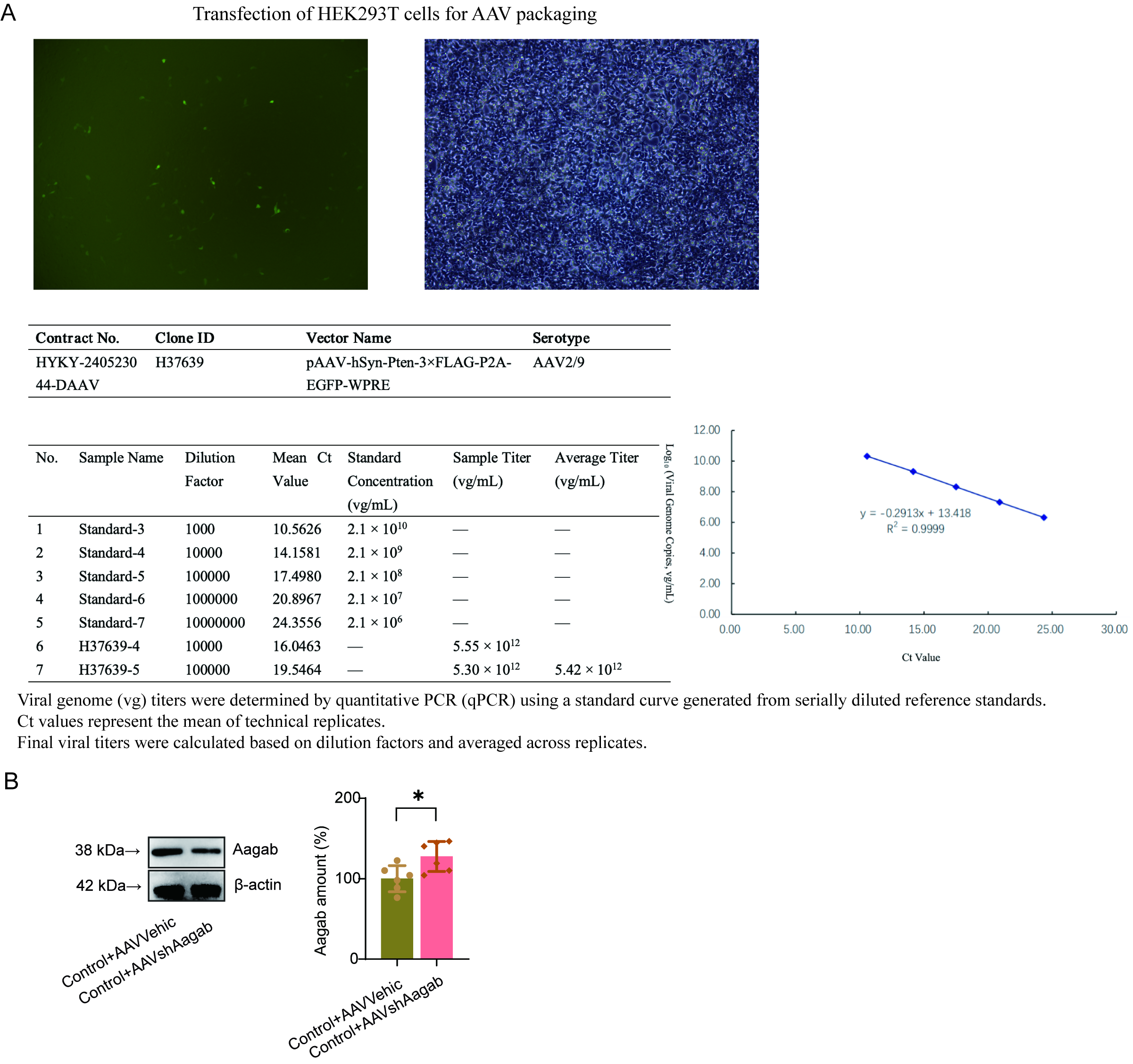

Supplement: Supplementary file 1 — Figure S1: cns70959‐sup‐0001‐FigureS1.tif. Validation of Viral Constructs. (A) Schematic illustration and titer validation of the AAVPTEN‐K13R construct. (B) Representative Western blot images and corresponding quantitative analysis showing protein knockdown efficiency of AAVshAagab (n = 10/group). [file CNS-32-e70959-s001.tif]
